# Pericarditis after SARS-CoV-2 Infection: Another Pebble in the Mosaic of Long COVID?

**DOI:** 10.3390/v13101997

**Published:** 2021-10-04

**Authors:** Francesco Carubbi, Alessia Alunno, Silvia Leone, Nicoletta Di Gregorio, Bernardina Mancini, Angelo Viscido, Rita Del Pinto, Sabrina Cicogna, Davide Grassi, Claudio Ferri

**Affiliations:** 1 Internal Medicine and Nephrology Unit, Department of Life, Health & Environmental Sciences, University of L’Aquila, 67100 L’Aquila, Italy; alessia.alunno82@gmail.com (A.A.); silvialeone.med@gmail.com (S.L.); nicolettadigregorio@yahoo.it (N.D.G.); bernardina.mancini@gmail.com (B.M.); angelo.viscido@univaq.it (A.V.); ritadelpinto@gmail.com (R.D.P.); davide.grassi@univaq.it (D.G.); claudio.ferri@univaq.it (C.F.); 2 Department of Medicine, ASL 1 Avezzano-Sulmona-L’Aquila, San Salvatore Hospital, 67100 L’Aquila, Italy; dipartimentomedicina@asl1abruzzo.it; 3 Cardiology and Coronary Care Unit, San Salvatore Hospital, 67100 L’Aquila, Italy

**Keywords:** SARS-CoV-2, long COVID, pericarditis

## Abstract

With the emerging success of the COVID-19 vaccination programs, the incidence of acute COVID-19 will decrease. However, given the high number of people who contracted SARS-CoV-2 infection and recovered, we will be faced with a significant number of patients with persistent symptoms even months after their COVID-19 infection. In this setting, long COVID and its cardiovascular manifestations, including pericarditis, need to become a top priority for healthcare systems as a new chronic disease process. Concerning the relationship between COVID-19 and pericardial diseases, pericarditis appears to be common in the acute infection but rare in the postacute period, while small pericardial effusions may be relatively common in the postacute period of COVID-19. Here, we reported a series of 7 patients developing pericarditis after a median of 20 days from clinical and virological recovery from SARS-CoV-2 infection. We excluded specific identifiable causes of pericarditis, hence we speculate that these cases can be contextualized within the clinical spectrum of long COVID. All our patients were treated with a combination of colchicine and either ASA or NSAIDs, but four of them did not achieve a clinical response. When switched to glucocorticoids, these four patients recovered with no recurrence during drug tapering. Based on this observation and on the latency of pericarditis occurrence (a median of 20 days after a negative nasopharyngeal swab), could be suggested that post-COVID pericarditis may be linked to ongoing inflammation sustained by the persistence of viral nucleic acid without virus replication in the pericardium. Therefore, glucocorticoids may be a suitable treatment option in patients not responding or intolerant to conventional therapy and who require to counteract the pericardial inflammatory component rather than direct an acute viral injury to the pericardial tissue.

## 1. Introduction

Severe acute respiratory syndrome coronavirus 2 (SARS-CoV-2) infection is characterized by a heterogeneous clinical picture ranging from absence of symptoms to the development of coronavirus disease 2019 (COVID-19) with varying degrees of severity [1]. Severe to critical COVID-19 encompasses respiratory failure, septic shock, and/or multiple organ dysfunction/failure; of note, immune mechanisms are critically involved in evolution of severe COVID-19, suggesting potential efficacy of immunomodulatory treatments [2]. The mortality rate varies according to age being less than 5% up to the 6th decade, and progressively increases by each single year of age in people aged 60 or above [3,4].

As time passes since the start of the pandemic, evidence on long-term outcomes of SARS-CoV-2 infection accrues. COVID-19 symptoms and complications may persist for months after the primary infection. Therefore, the term “long COVID” was coined. According to the National Institute for Health and Care Excellence (NICE), long COVID includes ongoing signs and symptoms of COVID-19 for 4 to 12 weeks following the acute infection and/or the persistence or development of signs and symptoms beyond 12 weeks from the onset of COVID-19 infection [5]. Interestingly, a total of 558 (13.3%) patients reported symptoms lasting ≥28 days after SARS-CoV-2 infection in a UK study [6], while Huang et al. found 76% of patients had at least 1 persistent symptom of COVID-19 at a mean of 186 days after hospital discharge [7].

Cardiovascular complications, in particular, myocardial injury/myocarditis, thromboembolic events, heart failure and cardiomyopathy, arrhythmias, acute coronary syndromes, and pericardial involvement, were described during the course of COVID-19 [8]. Unfortunately, however, little is known about cardiovascular manifestations occurring after clinical and virologic recovery from SARS-CoV-2 infection. Therefore, it is unclear whether long COVID also encompasses cardiovascular manifestations and to what extent [9]. With regard to myocardial changes following SARS-CoV-2 infection, myocardial oedema and late gadolinium enhancement were observed in 58% of 26 Chinese patients presenting with long COVID cardiac symptoms [10]. A myocarditis-pattern was observed up to 30 days after SARS-CoV-2 infection in the 27% of 148 patients in the UK who had myocardial injury [11]. Data on pericardial involvement are scarce, and a recent systematic literature review (SLR) including studies on adult patients undergoing any type of cardiac assessment after COVID-19 recovery reported a prevalence of pericardial enhancement in 63/758 patients and of pericardial effusion in 99/758 patients (on cardiac magnetic resonance (CMR)) or 12/811 patients (on echocardiography) [12]. Unfortunately, only four studies on CMR included in the SLR reported a formal clinical diagnosis of myopericarditis (15/785 patients) and pericarditis (4/785 patients), while only one study using echocardiography reported a formal clinical diagnosis of pericarditis [13]. Based on these results, small pericardial effusion may be relatively common in the postacute period of COVID-19, but overt pericarditis, especially with symptoms, seems a rarer manifestation. Direct virus-mediated cytotoxicity, ACE 2 receptor downregulation, immune-mediated inflammation affecting myocardium and pericardium and an inflammatory response are the most common underlying mechanisms postulated for the long COVID cardiovascular manifestations [14].

The knowledge gap regarding post-COVID cardiovascular manifestation may raise important clinical dilemmas in physicians dealing with patients with new onset symptoms such as chest pain/tightness, palpitations, dizziness, and an increase in resting heart rate who also report a previous SARS-CoV-2 infection. These symptoms in fact may appear in both hospitalized and nonhospitalized patients, and there is no clear relationship with pre-existing cardiovascular diseases [15].

Here, we describe a series of patients who developed pericarditis after clinical and virological recovery from SARS-CoV-2 infection.

## 2. Materials and Methods

### Patients

Cases were recorded prospectively following admission either to the ward or the outpatient clinic of the Internal Medicine and Nephrology Unit of the University of L’Aquila between January and June 2021. Inclusion criteria were limited to a previous diagnosis of SARS-CoV-2 infection and a diagnosis of overt pericarditis at the time of referral to our Unit. The severity of COVID-19 at the time of infection ascertainment was defined according to the WHO clinical progression scale [16]. This is a 10-point scale with the following structure: 0 = uninfected; 1 = asymptomatic infection; 2 = mild symptomatic disease (no assistance needed); 3 = mild symptomatic disease (assistance needed); 4 = hospitalized with moderate disease not requiring oxygen therapy; 5 = hospitalized with moderate disease requiring oxygen by mask or nasal prongs; 6 = hospitalized with severe disease requiring oxygen by non-invasive ventilation or high flow; 7 = hospitalized with severe disease requiring intubation and mechanical ventilation (MV) with a partial pressure of oxygen (p0_2_)/fraction of inspired oxygen (Fi0_2_) ≥ 150 or oxygen saturation (Sp0_2_)/ Fi0_2_ ≥ 200; 8 = MV with p0_2_/Fi0_2_ < 150 (Sp0_2_/Fi0_2_ < 200) or vasopressors; 9 = MV with p0_2_/Fi0_2_ < 150 and vasopressors, dialysis, or extracorporeal membrane oxygenation; 10 = dead. Details obtained from the medical record included demographics, past medical history, laboratory investigations (including full blood count and C-reactive protein), radiological findings (chest X-ray and trans-thoracic echocardiography), clinical management, patient progress, and survival. Patient data are presented as absolute values, percentages, and median (range). This study was conducted in compliance with the protocol of Good Clinical Practices and the Declaration of Helsinki.

## 3. Results

Seven patients with pericarditis and a positive history of SARS-CoV-2 recovered infection are described (Table 1). Three patients (43%) were males, 4 (57%) were females, and the median age was 61 years (range 52–75 years). Two patients (28%) had no comorbidity, while the other 5 patients had between 1 and 5 comorbidities. All patients had a previous polymerase chain reaction (PCR)-proven SARS-CoV-2 infection with clinical features ranging from mild–moderate (6 patients, 86%) to severe disease (1 patient, 14%). Two patients with WHO scale = 2 received no treatment for COVID-19, 3 patients with WHO scale =3 were treated at home with glucocorticoids + azithromycin and 2 of them also low-molecular-weight heparin (Table 2). The 2 hospitalized patients received glucocorticoids and low-molecular-weight heparin, and the patient with the more severe clinical features (WHO scale = 6) also received remdesivir. All patients fully recovered from SARS-CoV-2 infection and the symptoms suggestive of pericarditis occurred at a median of 20 days (range 15–28 days) after the negative nasopharyngeal swab. All patients had chest pain, and 4 patients (57%) also complained dyspnea, palpitations, and fever. Two patients (28%) also reported arthralgia. Pericardial effusion was detected by echocardiography in all patients and the 4 patients with a broader spectrum of symptoms also displayed pleural effusion at chest X-ray. Electrocardiographic abnormalities were detected in 3 patients, 2 of which had PR depression and 1 had ST elevation. The patient with ST elevation and one of those with PR depression also showed a mildly increase of troponin-I levels. Although the patients did not undergo CMR assessment, echocardiography did not identify signs of myocarditis. These electrocardiographic/laboratory findings without clear myocardial involvement on echocardiography, may suggest a possible isolated inflammation of the epicardium [17]. We excluded specific identifiable causes of pericarditis including infectious, autoimmune, neoplastic, and metabolic diseases alongside iatrogenic and traumatic causes. We also excluded, aortic dissection, pulmonary arterial hypertension, and chronic heart failure. Treatment was started with colchicine 1 mg/day in combination with either ibuprofen or acetylsalicylic acid (ASA) [17], as detailed in Table 3. Three patients recovered over a median of 5 weeks (range 5–7 weeks) without need of hospital admission. Four patients (57%) required hospitalization after a median of four weeks (range 3–6 weeks) due to inefficacy or intolerance (the latter mainly to colchicine) of the treatment and upon admission prednisone 0.5 mg/kg/day was started. Tapering and withdrawal of the steroid therapy occurred over a median of 6 weeks (range 6–8 weeks). No complication and/or recurrences occurred during follow up and at last contact (median 9 weeks after pericarditis onset) all patients remained asymptomatic with no signs of pericarditis.

## 4. Discussion

Although cardiovascular system involvement is common during SARS-CoV-2 infection, data on cardiovascular manifestations after the recovery from COVID-19 are scarce [9].

Here, we reported a series of 7 patients developing pericarditis after a median of 20 days from clinical and virological recovery from SARS-CoV-2 infection. We excluded specific identifiable causes of pericarditis hence we speculate that these cases can be contextualized within the clinical spectrum of long COVID.

The incidence of acute pericarditis was reported as 27.7 cases per 100,000 population per year in an Italian urban area [18]. Acute pericarditis is an inflammatory pericardial syndrome with or without pericardial effusion, and the clinical diagnosis is based on well-defined criteria [17]. Recurrence affect approximately 30% of patients within 18 months after a first episode of acute pericarditis [19,20]. This disease has a wide etiological spectrum but a specific cause is often not identified, and the diagnosis of idiopathic cases is essentially a diagnosis of exclusion, supported by the presence of ≥2 of the following: chest pain, pericardial friction/rub, electrocardiographic changes, and pericardial effusion without evidence of underlying infectious or noninfectious causes [21,22]. However, in the management of pericardial syndromes, a major controversy is the role of an extensive aetiological investigation and admission for all patients with pericarditis or pericardial effusion [17,23]. In particular, an Italian study from 2004 noted that viral etiology is commonly presumed but very often not confirmed because it constitutes a non-negligible and rather time-consuming expense. It was suggested that in the absence of risk factors for severe pericarditis, neither the performance of tests to establish a specific etiological diagnosis nor hospitalization of the patient is necessary in most cases [24]. Most cases of acute pericarditis in developed countries are believed to be secondary to either viral infections or are autoreactive [17,25,26,27] and acute viral pericarditis often presents as a self-limited disease, with most patients recovering without complications [23]. Cardiotropic viruses can directly cause pericardial and/or myocardial inflammation via two mechanisms: (i) direct cytolytic or cytotoxic effects (in particular enteroviruses); (ii) via T and/or B cell–driven immune-mediated mechanisms (in particular herpesviruses). Moreover, the persistence of viral nucleic acid without virus replication in the pericardium and/or myocardium can sustain the ongoing inflammation and effusions via autoimmune processes directed against specific cardiac proteins by molecular mimicry [25]. In addition, the so-called postcardiac injury syndromes are presumed to have an autoimmune pathogenesis triggered by initial damage to pericardial and/or pleural tissues [28].

Cardiac involvement secondary to SARS-CoV-2 infection is based on its mechanism of infecting human cells by binding to the transmembrane ACE-2 [29], and, probably, on the reduced innate antiviral defense against a novel virus [30]. However, specific immune mechanisms that occur in cardiac involvement, including pericarditis, after SARS-CoV-2 infection recovery remain unknown. It is reasonable to postulate that an insufficient or delayed humoral response may decrease the virus clearance locally at the peri-myocardium, and that local persistence of viral material would increase both tissue-homing of inflammatory cells and release of inflammatory molecules. In this context, molecular mimicry can result in the production of autoantibodies against cardiac proteins, leading to a cardio-specific autoimmune response that enhances this cardiac inflammation. The immune-mediated pathogenesis is supported by a latent period of a few weeks until the appearance of the first manifestations and the response to anti-inflammatory drugs (nonsteroidal anti-inflammatory drug (NSAIDs), corticosteroids, colchicine).

Concerning the relationship between COVID-19 and pericardial diseases, pericarditis appears to be common in the acute infection but rare in the postacute period of COVID-19, while small pericardial effusions may be relatively common in the postacute period of COVID-19. In hospitalized patients with COVID-19, diffuse acute ST changes consistent with pericarditis were present in 12% of subjects [31]. Clark and colleagues found only 1 case of pericarditis in an imaging study of 59 collegiate athletes [32]. Pericarditis was present in just 0.3% of competitive athletes systematically screened after COVID-19 [33]. In the postacute period of COVID-19, a pericardial effusion was identified using CMR in a proportion of patients ranging from 5 to 20% [11,34].

Cardiovascular morbidities are critically important to recognize and tackle during SARS-CoV-2 acute infection. Reducing these complications in addition to the primary pulmonary injury, drastically reduces short-term mortality. However, it is challenging to predict and promptly diagnose long-term cardiovascular manifestations related to SARS-CoV-2 infection. One of the first studies in this field concluded that 78% of recovered adult patients (*n* = 100; median age, 49 years) had ongoing cardiac involvement, of whom 71% had detectable high-sensitivity troponin T and 60% had myocardial inflammation based on interpretation of findings from CMR imaging [34]. This alarmingly high prevalence of imaging abnormalities suggestive of myocardial injury and inflammation deserves to be discussed. Firstly, it is unclear how this elevated prevalence compares with populations recovering from other viral infections or acute illnesses because troponin measurements and CMR examinations are not routinely obtained in these settings. Secondly, CMR imaging should be interpreted with caution in the absence of baseline pre–SARS-CoV-2 infection values, serial follow-up, and robust pathologic correlation. Finally, the finding that 60% of patients have myocardial inflammation is >10 times higher than the total of current autopsy data, in which only 6 cases of myocarditis were noted from 165 individuals across 12 studies [9,35]. Based on autopsy studies, myocarditis in acute COVID-19 has a lymphocytic predominance, and viral inclusions are rarely seen, suggesting myocarditis in acute COVID-19 is a sequela of an inflammatory response rather than a phenomenon due to direct viral attack [36].

In this regard, the treatment and clinical evolution of our 7 cases may shed some light on the matter. Aspirin, NSAIDs, and colchicine are the mainstay of pericarditis treatment, and many patients fully respond with no need for additional treatment [17]. In general, limited use of glucocorticoids is recommended, particularly if the etiology is unknown, since corticosteroid therapy is an independent risk factor for recurrence [37]. A possible explanation for this finding is that corticosteroids might impair virus clearance [38]. Conversely, glucocorticoids are effective, and therefore recommended, in pericarditis related to autoimmune diseases (e.g., systemic lupus erythematosus) owing to the different pathogenic mechanism [17]. All our patients were treated with a combination of colchicine and either ASA or NSAIDs but 4 of them did not achieve a clinical response. When switched to glucocorticoids, all patients recovered with no recurrence during drug tapering. Based on this observation and on the latency of pericarditis occurrence (a median of 20 days after a negative nasopharyngeal swab), it is tempting to speculate that post-COVID pericarditis may be linked to ongoing inflammation sustained by the persistence of viral nucleic acid without virus replication in the pericardium. Therefore, glucocorticoids may be a suitable therapeutic option in patients not responding or intolerant to conventional therapy and requiring treatment to counteract the pericardial inflammatory component rather than directed at an acute viral injury to the pericardial tissue [39].

Data on risk factors for developing long COVID are scarce and controversial. Risk factors seem to include female gender, higher number of pre-existing medical conditions, in particular, arterial hypertension, obesity, asthma, psychiatric condition, or an immunosuppressive condition [6,40]. Of interest, and in line with these observations, the only two patients in our cohort without comorbidities were females, underlying the relevance of female gender as independent risk factor for long COVID regardless of comorbidities.

As far as age was concerned, the group most greatly affected by long COVID symptoms is 35–49 years, followed by 50–69 years, and the ≥70 years group [41]. Interestingly, a cross sectional study showed that a more severe acute phase of COVID-19 may transform into the development of more severe symptoms of long COVID [42].

## 5. Conclusions

With the emerging success of the COVID-19 vaccination programs, the incidence of acute COVID-19 is decreasing. However, given the high number of people who contracted SARS-CoV-2 infection and recovered, we will be faced with a significant number of patients with persistent symptoms even months after their COVID-19 infection. In this setting, long COVID and its cardiovascular manifestations, including pericarditis, need to become a top priority for healthcare systems, as a new chronic disease process. Although our study displays some limitations such as the lack of incidence data of post-COVID pericarditis and the lack of CMR assessment, our case series provides important information in the field worth further investigation in larger studies.

A multidisciplinary cooperation and larger controlled studies are needed to reliably estimate and define an evidence-based approach to the long-term cardiovascular manifestations of SARS-CoV-2 infection. This should include the identification of patients at increased risk of these complications and of effective strategies to prevent and treat this condition. Finally, post-mortem analyses not only of patients deceased during acute COVID-19, but also of patients who recovered from the infection and subsequently deceased from cardiovascular diseases may shed additional light the pathobiology of SARS-CoV-2 and its relationship with cardiovascular manifestations.

## Figures and Tables

**Table 1:** Demographic characteristics and comorbidities of patient cohort.

ID	Age (Years)	Gender	Comorbidities							
			*SAH*	*AF*	*OA*	*OP*	*Obesity*	*Dyslipidemia*	*Depression*	*Hypothyroidism*
#1	75	M	x		x					
#2	73	F	x		x	x				x
#3	52	M					x			
#4	69	M	x	x	x		x	x		
#5	42	F								
#6	45	F								
#7	61	F	x					x	x	

SAH, systemic arterial hypertension; AF, atrial fibrillation; OA, osteoarthritis; OP, osteoporosis.

**Table 2:** Characteristics and management of SARS-CoV-2 infection and timing of pericarditis symptom occurrence.

ID	WHO Scale ^1^	Management of COVID-19					Days between the First Positive NP Swab and Pericarditis Symptoms	Days between the First Negative NP Swab and Pericarditis Symptoms
		*AZT*	*GC*	*LMWH*	*RDV*	*Oxygen*		
#1	2						25	15
#2	3	x	x				32	18
#3	3	x	x	x			50	21
#4	5		x	x		x	45	21
#5	2						31	15
#6	3	x	x	x			42	20
#7	6		x	x	x	x ^2^	54	28

^1^ From Lancet Infect Dis 2020;20: e192–97; ^2^ high-flow oxygen therapy. AZT, azithromycin; GC, glucocorticoids; LMWH, low-molecular weight heparin; RDV, remdesivir; NP, naso-pharingeal; WHO, World Health Organization.

**Table 3:** Management and evolution of pericarditis.

ID	1st Line Therapy					2nd Line Therapy	Last Follow-up ^1^	
	*ASA 1 g/d*	*NSAIDs IBP 1,800 mg/d*	*COL 1 mg/d*	*Response*	*Timepoint (weeks)*	*PDN 0.5 mg/kg/d*	*Symptoms and/or signs*	*Timepoint (weeks)*
#1		x	x	No	3	x	None	9
#2	x		x	No	4	x	None	10
#3		x	x	Yes	3		None	5
#4		x	x	No	4	x	None	10
#5	x		x	Yes	4		None	7
#6		x	x	Yes	3		None	5
#7	x		x	No	6	x	None	14

ASA, acetylsalicylic acid; NSAIDs, nonsteroidal anti-inflammatory drugs; IBP, ibuprofen; COL, colchicine; PDN, prednisone; d, day. ^1^ upon completion of therapy tapering.

## Data Availability

All data relevant to the study are included in this article.
